# Sequence variant at 8q24.21 associates with sciatica caused by lumbar disc herniation

**DOI:** 10.1038/ncomms14265

**Published:** 2017-02-22

**Authors:** Gyda Bjornsdottir, Stefania Benonisdottir, Gardar Sveinbjornsson, Unnur Styrkarsdottir, Gudmar Thorleifsson, G. Bragi Walters, Aron Bjornsson, Ingvar H. Olafsson, Elfar Ulfarsson, Arnor Vikingsson, Ragnheidur Hansdottir, Karl O. Karlsson, Thorunn Rafnar, Ingileif Jonsdottir, Michael L. Frigge, Augustine Kong, Asmundur Oddsson, Gisli Masson, Olafur T. Magnusson, Tomas Gudbjartsson, Hreinn Stefansson, Patrick Sulem, Daniel Gudbjartsson, Unnur Thorsteinsdottir, Thorgeir E. Thorgeirsson, Kari Stefansson

**Affiliations:** 1 deCODE Genetics/Amgen, Inc., Reykjavik IS-101, Iceland; 2 Department of Neurosurgery, Landspitali University Hospital, Reykjavik IS-101, Iceland; 3 Faculty of Medicine, University of Iceland, Reykjavik IS-101, Iceland; 4 Department of Medicine, Landspitali, National University Hospital, Reykjavik IS-101, Iceland; 5 Thraut Fibromyalgia Clinic, Reykjavik IS-101, Iceland; 6 Department of Dentistry, University of Iceland, Reykjavik IS-101, Iceland; 7 School of Engineering and Natural Sciences, Science Institute, University of Iceland, Reykjavik IS-101, Iceland; 8 Department of Surgery, Landspitali University Hospital, Reykjavik IS-101, Iceland

## Abstract

Lumbar disc herniation (LDH) is common and often debilitating. Microdiscectomy of herniated lumbar discs (LDHsurg) is performed on the most severe cases to resolve the resulting sciatica. Here we perform a genome-wide association study on 4,748 LDHsurg cases and 282,590 population controls and discover 37 highly correlated markers associating with LDHsurg at 8q24.21 (between *CCDC26* and *GSDMC*), represented by rs6651255[C] (OR=0.81; *P*=5.6 × 10^−12^) with a stronger effect among younger patients than older. As rs6651255[C] also associates with height, we performed a Mendelian randomization analysis using height polygenic risk scores as instruments to estimate the effect of height on LDHsurg risk, and found that the marker's association with LDHsurg is much greater than predicted by its effect on height. In light of presented findings, we speculate that the effect of rs6651255 on LDHsurg is driven by susceptibility to developing severe and persistent sciatica upon LDH.

## Abstract (web summary)

Lumbar disc herniation (LDH) can cause persistent sciatica, and in some cases surgery is required to relieve symptoms. Here, the authors carry out a genome-wide association study using microdiscectomy as an indicator of severe LDH, and find a locus on chromosome 8 associated with this condition.

Sciatica describes the often debilitating form of neuropathic pain that radiates along the sciatic nerve (radiculopathy)^1^. While sciatica can be of various etiologies, the majority of cases (85–90%) are caused by lumbar disc degeneration (LDD) resulting in herniation of disc material from the nucleus pulposus into the epidural space, leading to mechanical and/or chemical insult to affected nerve roots^1^,^2^,^3^.

The pathophysiology of lumbar disc herniation (LDH) is not completely understood^4^. Imaging studies of different populations have shown that LDD starts in early adolescence and affects the majority of people over the course of their life^5^,^6^,^7^. Therefore, drawing a diagnostic line between normal consequences of aging and pathological disc changes remains challenging^5^. Twin studies suggest that genetics strongly influence various radiological signs of LDD with heritability estimated up to 74% (95% confidence interval (CI) 64–81%)^8^,^9^. However, the search for genetic risk factors has not yielded conclusive results^10^,^11^. A recent genome-wide association study (GWAS), utilizing a quantitative measure created from radiological signs of LDD (lumbar disc space narrowing and presence of osteophytes) in 4,600 individuals of European descent, uncharacterized for LDD symptoms, identified four single-nucleotide polymorphisms (SNPs) with *P* between 1.8 × 10^−9^ and 3.3 × 10^−8^ (effect (*β)* between −0.13 and 0.23)^12^.

Although herniated lumbar discs are the most common cause of sciatica, they are also observed in up to 50% of asymptomatic adults by magnetic resonance imaging^13^,^14^,^15^. Furthermore, about a third of symptomatic LDH resolve within weeks or months regardless of intervention^13^,^16^,^17^ with only a fraction (5–10%) leading to persistent sciatica or radiculopathy requiring surgery^18^,^19^. Microdiscectomy, a minimally invasive spinal surgery for removal of herniated lumbar disc tissue, is indicated only for individuals with severe, progressive and persistent sciatica symptoms that are consistent with location of the radiologically confirmed herniated disc^13^,^20^. These surgical criteria, therefore, define a clear and specific phenotype of sciatica secondary to LDH pathology that we here show associates strongly with a sequence variant at 8q24.21 between *GSDMC* and *CCDC26.* Our results provide novel insights into the biological underpinnings of this painful condition.

## Results

### A sequence variant at 8q24.21 associates with LDHsurg

We performed a GWAS of 4,748 Icelanders who underwent microdiscectomy of a herniated lumbar disc (LDHsurg) and 282,590 population controls. The sample represents 90% of those undergoing this surgical procedure in Iceland over an 18 year period or from 1997–2015 (Table 1).

**Figure 1:**
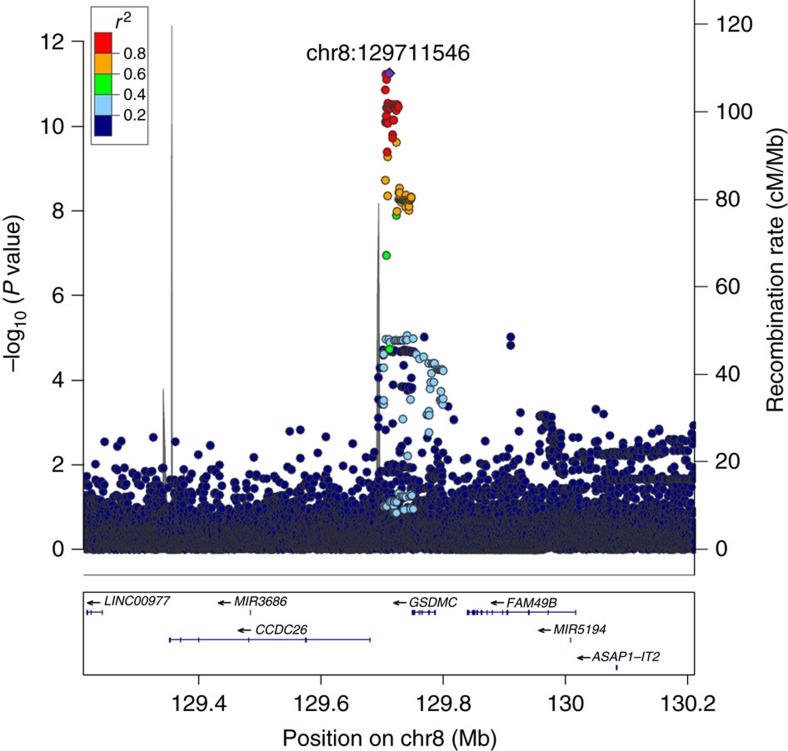
Regional association plot for the 8q24.21 intergenic locus that associates with undergoing LDHsurg (*N*=4,748). *P* values (−log10) of SNP association with LDHsurg in Iceland is plotted against their positions at the 8q24.21 locus. SNPs are coloured to reflect their LD with rs6651255 (chr8:129711546) (purple diamond) in the data set. The right *y* axis shows calculated recombination rates at the chromosomal location, plotted as solid grey lines. Known genes in the region are shown underneath the plot, taken from the UCSC genes track in the UCSC Genome Browser. All positions are in in NCBI Build 38 coordinates. A *χ*2-test was used to calculate *P* values.

We applied genome-wide significance thresholds weighted for variant classes, using the threshold of *P<*1.1 × 10^−9^ assigned to variants in the lowest impact functional class^21^ (Methods). Altogether, 37 highly correlated sequence variants (*r*^*2*^ with top marker >0.78) associating with LDHsurg were identified, located at 8q24.21 between *GSDMC* and *CCDC26* and spanning ∼20 kb (chr8:129,705,470–129,726,726, hg38) (Fig. 1, Supplementary Fig. 1 and Supplementary Table 1). The most significant association was with rs6651255[C] (AF=23%; odds ratio (OR)=0.81; 95% CI=0.77, 0.86; *P*=5.6 × 10^−12^, a *χ*^2^-test was used to calculate *P* values for all GWAS associations) (Methods). Conditional analysis showed that the signal of 37 variants is fully accounted for by rs6651255[C] (all adjusted *P*≥0.15) and no other markers within±2 Mb reached significance. The effect of rs6651255 on LDHsurg did not differ by gender (male vs female *P*=0.85). A significant difference in effects was, however, observed when comparing those who underwent LDHsurg at an earlier age (≤40 years) to those aged 41 years and older at first LDHsurg (*P*=1.8 × 10^−3^) (Table 2).

### The LDHsurg signal at 8q24.21 does not associate with cancer

The 8q24.21 region, containing *MYC* and *PVT1,* is known to host a number of variants associating with risk of several cancer types^22^,^23^,^24^,^25^. However, that region is about 2 Mb upstream of rs6651255. None of the reported markers for cancer in the 8q24.21 region correlate with the LDHsurg signal. Searching for associations of rs6651255[C] with several common cancers^26^ revealed no significant association once the number of tests had been taken into account. The closest protein coding gene (∼370 kb downstream) is *GSDMC*, a gene initially found by researchers seeking genes upregulated in metastatic mouse melanoma cells (hence also termed *MLZE*)^27^. Besides expression in skin epithelium, *GSDMC* is primarily expressed in trachea and spleen and although its function is largely unknown, as a member of the Gasdermin superfamily, it is thought to be involved in regulation of epithelial cell development, apoptosis, carcinogenesis and immune-related functions^28^,^29^,^30^,^31^.

Approximately 30 kb upstream of rs6651255 is the full-length mRNA encoding *CCDC26*. Also known as *RAM*, *CCDC26* encodes a retinoic acid-dependent modulator of myeloid differentiation^32^,^33^. Retinoic acid is involved in regulation of skeletal growth, chondrocyte proliferation and aggrecan (the major proteoglycan component of the intervertebral disc) expression and content^34^. Indeed, *CCDC26* was considered a candidate gene in a GWAS of cleft lip showing a strong association with a variant (rs987525) located ∼400 kb upstream of *CCDC26* (ref. 35). This cleft lip variant is 800 kb upstream of rs6651255 (*r*^*2*^=0.00056, *D*′=0.084) and in the present study showed no association with LDHsurg in the Icelandic sample (OR=0.98, *P*=0.40). Including rs987525, there are further four SNPs listed in the GWAS catalogue (URLs) located in or near *GSDMC* or *CCDC26*. They are rs4295627 associating with glioma, rs10098310 with monocyte count and rs4733724 and rs6470764 with height. Testing these variants in corresponding Icelandic phenotypes shows that SNPs in this region behave comparably in Icelandic data as in other populations (Supplementary Table 2).

### The effects of rs6651255 on LDHsurg and height

Three out of the 37 correlated SNPs representing the LDHsurg signal have previously been reported to associate with adult human height in large meta-analyses^36^,^37^,^38^ (Supplementary Tables 2 and 3). All are highly correlated with rs6651255 (*r*^*2*^>0.9 in Iceland). Consistently, rs6651255[C] associating with lower risk of LDHsurg, also associates with less height in the Icelandic data (*β*=−0.03 s.d. (corresponding to −0.2 cm), *P*=2.2 × 10^−5^; *N*=88,835, *χ*^2^ test). However, neither rs6470764[T] representing rs6651255, nor any of the 16, out of the 37 correlated SNPs of the signal that were tested in the GIANT BMI meta-analysis^39^, associate with BMI (lowest *P* value of 0.10 (*β*=0.007; s.e.=0.004; *N*=321,563)).

Consistent with previous epidemiological results^40^, we observed a positive association between adult height and risk of LDHsurg in Icelandic patients (OR=1.25 for 10 cm increase in height; *P*=4.6 × 10^−10^; *N*_case_=1,929; *N*_control_=79,883, *t*-test) (Methods). To examine to what degree known adult height variants collectively explain the association with LDHsurg, we computed adult height polygenic scores, based on adjusted GWAS scores for 1.7 million markers tested in the GIANT study^37^ (excluding Icelandic data), for Icelanders who had been genotyped with various Illumina SNP chips (Methods). In our data the adult height polygenic scores significantly associate with LDHsurg case–control status (OR=1.36 for 10 cm increase in height; *P*=8.4 × 10^−6^; *N*_case_=3,085; *N*_control_=132,822, *t*-test).

**Figure 2:**
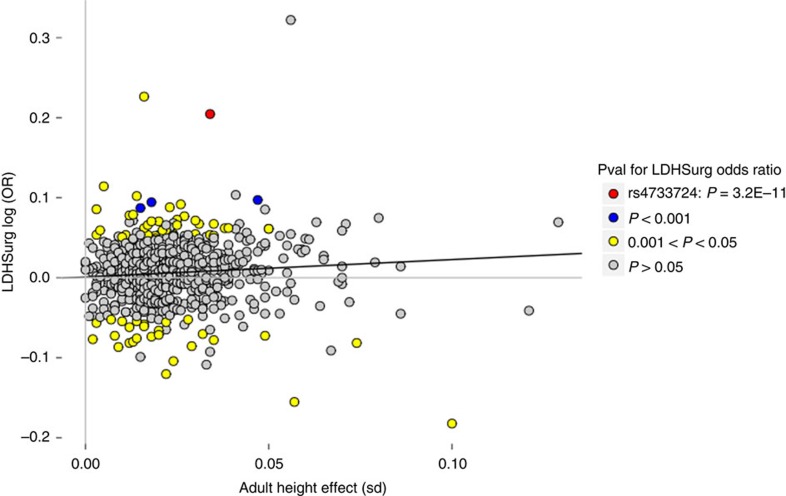
Correlation of GIANT variants' effect on height and risk of LDHsurg. Scatter plot showing how the 689 out of 697 adult height variants reported by GIANT^37^ that we have LDHsurg association results for, affect adult height (*x* axis) and LDHsurg (*y* axis) in our data. The effect allele (minor or major) of each variant was concluded to be the one that has a positive effect on adult height in our data. The black solid line, *y*=0.0010+0.22*x*+e, represents weighted linear regression, excluding rs4733724, with *R*^*2*^=0.0072 and *P*=0.026, using MAF(1-MAF) as weights. A *t*-test was used to calculate *P* value.

Given that adult height variants associate collectively with LDHsurg and a SNP corresponding to the LDHsurg signal (rs4733724, Supplementary Table 3) was among the 697 adult height variants reported by the GIANT meta-analysis^37^, we tested whether the other 696 GIANT variants associate independently with LDHsurg (Fig. 2, Methods). While Fig. 2 demonstrates a small positive correlation (*R*^*2*^=0.0072, *P*=0.026, *t*-test) between effects of these GIANT variants on height and logarithm of their LDHsurg risk (logOR), none of the other GIANT variants significantly associate with LDHsurg at a threshold of 7.2 × 10^−5^ (*P*<0.05/697). The SNP representing our top marker is a clear outlier (Fig. 2). Using the slope from the correlation plot in Fig. 2, the predicted effect of rs6651255[C] on LDHsurg, through its effect on height, would result in an odds ratio of 0.99, showing that the LDHsurg effect is not proportional to the height effect. These analyses clearly show that the effect of rs6651255 on LDHsurg greatly surpasses that expected due to its effect on height (Fig. 2).

### Characterizing rs6651255 effects on related phenotypes

In light of potential structural effects of rs6651255[C] implied by its height association, we also investigated the variant's effect on available phenotypes relating to vertebral bone structure in Icelandic data^41^. The variant did not associate with osteoarthritis of the spine (OR=0.96; *P*=0.35), bone mineral density of the spine (*β*=−0.01; *P*=0.31), or osteoporotic vertebral fractures (OR=1.02; *P*=0.64) (Supplementary Table 4).

As coded radiological reports for this large sample of LDHsurg cases were unavailable for the study, we tested all published markers (*P*<10^−5^) from the previously mentioned GWAS of radiologically defined LDD (a quantitative trait phenotype based on lumbar disc space narrowing and osteophytes growth)^12^ for association with our LDHsurg phenotype. None of the 44 markers associates with risk of undergoing LDHsurg (*P*<0.05/44=1.1 × 10^−3^, Supplementary Table 5).

To further characterize the effects of rs6651255, we identified an independent sample of Icelandic cases from hospital records (*N*_case_=930) diagnosed as the LDHsurg cases, (with intervertebral disc disorder with radiculopathy represented by ICD-10 code M51.1) who, however, had no history of LDHsurg. This indicating that their associated sciatica symptoms resolved over time and did not become severe enough to warrant surgery. There was no association observed with rs6651255 in this sample (OR=0.95; *P*=0.50).

Taken together, these findings suggest that the effect of rs6651255 on risk of undergoing LDHsurg is driven, not primarily by the morphology associated with herniated lumbar discs, but rather by the severity and persistence of associated sciatica. We therefore tested whether rs6651255[C] associated with chronic or neuropathic pain in independent Icelandic samples with fibromyalgia (*N*_case_=2,142), temporomandibular joint disorder (TMD, *N*_case_=1,455) and migraine (*N*_case_=3,816), in addition to a small sample with neuropathic pain as determined by the DN4 screening questionnaire (*N*_case_=394) (Methods). No significant associations with rs6651255 were detected. As larger sample sizes are needed to reach definite conclusions we provide the association results (Supplementary Table 6) to facilitate future meta-analysis efforts.

### Effect of rs6651255 on expression of nearby genes

Finally, to assess the potential effect of rs6651255 on gene expression (eQTL) we examined data from the Genotype-Tissue Expression (GTEx) project (URLs) available from multiple tissues, however, not including tissue from the intervertebral disc. In GTEx data, rs6651255 has a significant eQTL (*cis* window defined as ±1 Mb around gene transcript start site, false discovery rate<0.05) with *GSDMC* in two tissues; esophagus-mucosa (*P*=9.2 × 10^−14^) and Sun-exposed skin of lower leg (*P*=4.5 × 10^−10^), while association with *CCDC26* (*RP11-274M4.1*) was only present in esophagus-mucosa (*P*=3.7 × 10^−6^) (Supplementary Table 7). However, the best LDHsurg association signal (represented by rs6651255) and the best eQTL in the same tissues do not coincide. The variant rs4345520 has the most significant eQTL with *GSDMC* (esophagus-mucosa, *P*=3.0 × 10^−42^; Skin—Sun-exposed (lower leg), *P*=3.3 × 10^−28^) and rs10092783 the most significant eQTL with *CCDC26* (RP11-274M4.1) (esophagus-mucosa, *P*=6.7 × 10^−16^). These two variants are highly correlated (*r*^*2*^=0.82), while they are moderately correlated with rs6651255 (all *r*^*2*^<0.29). (Supplementary Table 7). In addition, a *cis*-eQTL analysis was performed in the Icelandic data based on RNA sequencing of white blood cells (*N*=1,002) and adipose tissue (*N*=673) samples. We assessed associations with probes from the five genes within ±500 Kb of rs6651255 (Fig. 1). At a significance threshold of 1.0 × 10^−2^ (*P*<0.05/5) we did not observe associations of rs6651255 with gene expression in white blood cells or adipose tissue.

## Discussion

We have discovered an association between common, correlated, intergenic sequence variants and risk of undergoing microdiscectomy of a herniated lumbar disc (LDHsurg); a spinal surgery performed to alleviate persistent and painful sciatica symptoms. The signal is represented by rs6651255[C] that also associates with less height, potentially reflecting structural effects involved in the pathogenesis of LDH. However, our results show that this variant does not affect risk of LDHsurg through height (Fig. 2). In addition, rs6651255 does not affect the skeletal phenotypes that we tested; osteoarthritis of the spine, bone mineral density of the spine or vertebral osteoporosis, nor did we find associations between markers from the only identified GWAS of morphological changes associated with LDD (albeit only radiologically defined without symptom characterization)^12^. Indeed, clinical studies have established that there is an unclear relationship between radiological signs of LDH and degree of sciatica or other clinical symptoms^15^. In keeping with the main indication for LDHsurg^13^,^20^; the neuropathic and painful symptoms of sciatica, we find of interest that height is inversely correlated with peripheral nerve conduction velocity^42^ and has been shown to increase risk of peripheral sensory neuropathy in diabetes^43^, peripheral insensate neuropathy^44^, HIV neuropathy^45^ and post-mastectomy neuropathic pain syndrome^46^. While the pathogenesis behind the relationship between height and neuropathic pain is unclear, increased height in humans is known to associate with increased axonal length and greater axon surface area^47^. The weak, but directionally consistent association (OR=0.83, *P*=0.04) of rs6651255[C] with neuropathic pain as measured by the DN4 is of interest in this context, but requires further study in larger samples.

When selecting cases for microdiscectomy, neurosurgeons have established that the herniated disc material is likely to cause the associated sciatica symptoms^13^,^20^. Indeed in most cases, rapid symptom relief follows microdiscectomy, but in up to 20% of cases symptoms persist after surgery^13^,^18^,^48^,^49^. Besides the symptoms caused by mechanical compression on nerve roots by the herniated lumbar disc, inflammatory and immunological consequences are also considered a factor in sciatica symptom severity and duration^50^,^51^. Of interest in this context is that *GSDMC*, downstream of rs6651255, is highly expressed in the spleen^27^ which plays a significant role in adaptive immune responses^52^. While the role of the immune system in regression of herniated discs and/or development of sciatica is not clear, the immune system is known to become activated by the otherwise immune-privileged nucleus pulposus as it herniates from its protected environment into the immune-regulated epidural space^53^. The subsequent migration of immune cells into the herniated disc tissues and increased expression of inflammatory cytokines is accompanied by the ingrowth of nociceptive nerve fibres into the disc, a process thought to contribute to the pathogenic development of the associated painful symptoms^53^.

The surgically defined phenotype used in this study was selected to represent a severe, symptomatic form of LDD, that is, herniated lumbar disc with sciatica indicating LDHsurg. In light of the clear clinical criteria associated with this type of surgery and the findings presented here, we speculate that the observed effect of rs6651255 on risk of undergoing LDHsurg is mainly driven by the severity of symptoms, that is, susceptibility to developing persistent and painful sciatica in the presence of the mechanical and biochemical nerve root insult created by a herniated lumbar disc. While our findings await replication in a comparable sample, we have provided compelling evidence for this variant having a significant role in the development of sciatica resulting from a herniated lumbar disc. The observed association in the Icelandic population is highly significant and we were able to exclude that the effect on sciatica is solely through the variant's effect on height. However, it is clear that further studies are required to complete this complicated but intriguing picture.

## Methods

### Study populations and phenotypes

Data on microsurgical excision of herniated lumbar intervertebral discs (LDHsurg) were obtained from the Landspitali National University Hospital (Landspitali) databases of surgical procedures (that is, lumbar microdiscectomy with surgical code ABC16 according to the NOMESCO (Nordic Medico-Statistical Committee) system)^54^. The so-defined phenotype is represented in the current paper by ‘LDHsurg'. Demographics of the defined sample are presented in Table 1. Landspitali is a tertiary reference centre hospital and the only hospital in Iceland offering neurosurgery. Microdiscectomy consists of removing a portion of the intervertebral disc, the herniated or protruding portion that is compressing the traversing spinal nerve root, by way of a small incision and using a surgical microscope. This well-established technique has become the most common surgery performed by neurosurgeons in Iceland and elsewhere^13^,^20^. It was first performed in Landspitali in 1981 and has since become standard treatment for herniated lumbar discs with persistent, painful sciatica or radiculopathy that does not respond to conservative treatment^20^. The data used in the present study cover microdiscectomy surgeries performed in the hospital's Department of Neurosurgery over a period of 18 years (1997–2015). As shown in Table 1, the proportions of genotyped cases out of the total cases undergoing microdiscectomy surgery during this period support non-bias in the discovery phenotype. Data on individuals diagnosed with herniated intervertebral discs with radiculopathy (ICD-10 code M51.1) who did not undergo microdiscectomy (LDHsurg), were obtained from the Landspitali database of discharge diagnoses (for the same time period as LDHsurg, or 1997–2015). During this period, M51.1 was the most prevalent hospital diagnosis preceding LDHsurg, with a total of 6,280 individuals with this diagnosis. The 5,288 individuals who underwent LDHsurg (Table 1) were excluded as were those diagnosed with M51.1 within 6 months of the end of the study period in 2015, to ascertain that we did not include newly diagnosed cases that would within 6 months become candidates for LDHsurg (according to clinical guidelines in Iceland, LDHsurg is performed if symptoms progress and/or severity persists for 2–3 months). Age and gender distribution of this sample of confirmed LDH without LDHsurg was comparable to the larger LDHsurg sample (Supplementary Table 8).

Height measurements from 88,835 Icelanders were collected in deCODE‘s resident assessment instrument studies. Height (cm) was either measured using a stadiometer with the subjects wearing no shoes or self-reported on questionnaires by individuals. Adult height measurements were corrected for year of birth and standardized separately for each of the sexes to have a standard normal distribution. Measured and self-reported heights were corrected separately. Height measurements were available for 1929 LDHsurg cases and 79,886 controls.

Information on vertebral osteoarthritis and bone mineral density was obtained from the Landspitali electronic health records. Secondary osteoarthritis and post-trauma osteoarthritis were excluded from the list. Bone densitometry (DEXA, Hologic QDR4500A) was also obtained from Landspitali^41^. Bone mineral density of the spine (L2-L4) was age and weight corrected and standardized in each gender separately. Vertebral fractures were self-reported in a detailed questionnaire of osteoporosis related information, using additional hospital data to increase classification accuracy, for example, by excluding high-trauma fractures, corticosteroid users, and women with early menopause from the fracture list^41^.

The Icelandic migraine sample was recruited from various sources including diagnoses from neurologists and self-reported migraine according to a validated questionnaire as has been described in detail elsewhere^55^. Fibromyalgia cases were characterized by a rheumatologist heading the only clinic in Iceland specializing in diagnosis and treatment of fibromyalgia. All patients were diagnosed according to the 2010 American College of Rheumatology (ACR) criteria^56^. Data on TMD were obtained via the dental department at the University of Iceland. All patients were diagnosed by dentists specializing in TMD following the diagnostic criteria for temporomandibular disorders (DC/TMD)^57^. Patients with a history of TMD secondary to trauma were excluded. Participants in the deCODE pain projects who answered the DN4 screening questionnaire for neuropathic pain^58^ were recruited on the basis of having various chronic pain conditions (chronic pain defined as lasting 3 months or longer) in addition to post-surgical patients who by the nature of their surgery had sustained neural lesions. On the basis of studies evaluating sensitivity and specificity of DN4 scores compared with clinical diagnoses of neuropathic pain, those who scored 4 and above on the DN4 scale of 0–7 were classified as having neuropathic pain^58^.

All samples and questionnaire data were collected through studies approved by the National Bioethics Committee and the Data Protection Authority in Iceland. Data were analysed within the context of a study on the genetics of chronic and neuropathic pain with permission from the National Bioethics Committee (VSNb2012090009/03.12) and overseen by the Data Protection Authority. All participants signed informed consent before blood samples were drawn. All personal identifiers were encrypted by a third-party system overseen by the Icelandic Data Protection Authority^59^. The association studies were performed using genotypes from these data in addition to whole-genome sequence data and imputation approaches previously outlined^60^ and further described below.

### Genotyping and imputation

Genotyping and imputation methods and the association analysis method in the Icelandic samples are previously described^60^ with some modifications described here. The whole genomes of 8,453 Icelanders were sequenced using Illumina technology to a mean depth of at least × 10 (median × 32). SNPs and indels were identified and genotypes called using joint calling with the Genome Analysis Toolkit HaplotypeCaller (GATK version 3.3.0) (ref. 61). The sequence variants identified in the 8,453 sequenced Icelanders were then imputed into 150,656 Icelanders who had been genotyped with various Illumina SNP arrays and their genotypes phased using long-range phasing^60^. Using familial imputation^60^, the sequence variants were imputed into untyped relatives of the array-typed to further increase the sample size for association analysis and increased the power to detect associations. Individuals who had undergone LDHsurg were either genotyped individuals (*N*=3,097) or untyped first and second degree relatives of genotyped individuals (*N*=1,651). The group of controls consisted of array-typed individuals (*N*=132,966) and untyped first and second degree relatives (*N*=149,624); resulting in 282,590 population controls. A total of 21.6 million variants were used in the association analysis under an additive model. All of the tested variants had imputation information over 0.85 and met quality control standards^60^.

### Sample preparation and DNA whole-genome sequencing methods

Our dataset contains samples obtained using three different library preparation methods from Illumina. In addition sequencing was performed using three different types of Illumina sequencing instruments.

- Standard TruSeq DNA library preparation method. Illumina GAIIx and/or HiSeq 2000 sequencers.
- TruSeq DNA PCR-free library preparation method. Illumina HiSeq 2500 sequencers.
- TruSeq Nano DNA library preparation method. Illumina HiSeq X sequencers.

The Standard TruSeq DNA library preparation method involves isolating approximately 1 μg of genomic DNA from frozen blood samples and fragmenting to a mean target size of ∼300–400 bp using a Covaris E210 instrument. The resulting fragmented DNA was end repaired using T4 and Klenow polymerases and T4 polynucleotide kinase with 10 mM dNTP followed by addition of an 'A' base at the ends using Klenow exo fragment (3′ to 5′-exo minus) and dATP (1 mM). Sequencing adaptors containing 'T' overhangs were ligated to the DNA products followed by agarose (2%) gel electrophoresis. Fragments of ∼450–500 bp were isolated from the gels (QIAGEN Gel Extraction Kit), and the adaptor-modified DNA fragments were PCR enriched for ten cycles using Phusion DNA polymerase (Finnzymes Oy) and a PCR primer cocktail (Illumina) needed for paired-end sequencing. Enriched libraries were purified using AMPure XP beads. The quality and concentration of the libraries were assessed with the Agilent 2100 Bioanalyzer using the DNA 1000 LabChip. Libraries were stored at −20 °C. Sequencing-by-synthesis (SBS) was performed on either Illumina GAII_x_ or HiSeq 2000 instruments, respectively. Paired-end libraries were sequenced using 2 × 76, 2 × 101 or 2 × 120 cycles of incorporation and imaging with Illumina SBS kits, TruSeq v5 for the GAIIx. For the HiSeq 2000, 2 × 101 cycles with SBS kits v2.5 or v3 were employed. Each library was initially run on a single lane on a GAII_x_ for validation, assessing optimal cluster densities, insert size, duplication rates and comparison with chip genotyping data. Following validation, the desired sequencing depth (× 10 to × 30) was then obtained using either sequencing platform. Targeted raw cluster densities ranged from 500–800 K^−1^ mm^−2^, depending on the version of both the sequencing chemistry and the data imaging/analysis software packages (SCS.2.8/RTA1.8 or SCS2.9/RTA1.9 for the GAIIx and HCS1.3.8. or HCS1.4.8 for HiSeq 2000). Real-time analysis involved conversion of image data to base calling in real-time.

Using the TruSeq DNA PCR-free library preparation method, paired-end libraries for sequencing were prepared according to the manufacturer's instructions (Illumina, TruSeq DNA PCR-free). In short, ∼1 μg of genomic DNA, isolated from frozen blood samples, was fragmented to a mean target size of 350 bp using a Covaris E210 ultrasonicator followed by clean-up using AmPure XP purification beads. Blunt-end DNA from the resulting fragments was generated using a mix of 3′>5′ exonuclease and 5′>3′ polymerase activities, respectively, followed by 5′-phosphorylation using T4 polynucleotide kinase. Size-selection of the blunt-end fragments was done using a two-step purification strategy with different ratios of the AmPure XP purification beads (× 0.6 and × 1). Finally, 3′-adenylation and ligation of barcoded adapters was performed, followed by clean-up with magnetic beads. The quality and concentration of the libraries were assessed with the Agilent 2100 Bioanalyzer using the DNA 1000 LabChip (Agilent). Barcoded libraries were stored at −20 °C. All steps in the workflow were monitored using an in-house laboratory information management system with barcode tracking of all samples and reagents. All samples were first pooled (12–24 plex) and sequenced on Illumina's MiSeq instruments (2 × 25 cycles) to assess quality and effective concentration of sequencing libraries. Subsequent deep sequencing was done on HiSeq 2500 instruments, were each sample was sequenced on three lanes, generating >100 Gb of raw data and at least 30X coverage. Sequencing was done using TruSeq v3 reagents, paired-end 2 × 100 cycles. System operation and base calling in real-time was done using HCS 2.2.38 and RTA 1.18.61.

Finally, the TruSeq Nano DNA library preparation method involves sample preparation and sequencing using a method that is essentially the same as described above for the TruSeq DNA PCR-free method, except the input amount was 100 ng of genomic DNA (instead of 1 μg) and following clean-up of adapter ligated DNA, the samples were enriched by eight-cycles of PCR using a PCR primer cocktail, followed by Ampure XP bead clean-up. The quality and concentration of the libraries were assessed with the Perkin Elmer LabChip GX instrument using the HT DNA HiSens reagent kit. Sequencing was done using the HiSeq X HD reagent kit. Each sample was loaded onto the HiSeq X instrument at a concentration of 300 pM and sequenced to high depth (>30 × ). System operation and base calling in real-time was done using HCSX 3.1.26 and RTA2 2.3.9.

### Association analysis

Association testing for case–control analysis was performed using logistic regression, adjusting for gender, age and county. A generalized form of linear regression was used to test for association of quantitative traits with SNPs.

Our method of testing for association takes the closest relatedness into account using a mixed effect model^60^. To further account for inflation in test statistics due to relatedness and stratification, we applied the method of linkage disequilibrium (LD) score regression^62^. With a set of 1.1M variants we regressed the χ^2^ statistics from our GWAS scan against LD score and used the intercept as a correction factor. The LD scores were downloaded from an LD score database (URL's). The estimated correction factor was 1.15 for LDHsurg, 1.05 for LDHsurg ≤40yrs, 1.08 for LDHsurg >40yrs, 1.08 for LDHsurg males and 1.06 for LDHsurg females. The correction factor was 1.48 for adult height, 1.07 for osteoporosis with vertebral fractures, 1.44 for osteoarthritis of the spine, 1.14 for bone mineral density of spine, 1.01 for LDH (ICD-10 code M51.1) excluding LDHsurg, 1.09 for TMD, 1.11 for fibromyalgia, 1.51 for migraine and 1.04 for DN4-determined neuropathic pain.

The threshold for genome-wide significance was corrected for multiple testing with a weighted Bonferroni adjustment using as weights the enrichment of variant classes with predicted functional impact among association signals^21^. With 21,568,490 sequence variants being tested, the weights given in the study by Sveinbjornsson *et al*.^21^ were rescaled to control the family-wise error rate. This yielded significance thresholds of 3.7 × 10^−7^ for high-impact variants (*N*=6,308), 7.4 × 10^−8^ for moderate-impact variants (*N*=121,793), 5.3 × 10^−9^ for low-impact variants (*N*=1,329,163), 3.4 × 10^−9^ for other DNase I hypersensitivity sites (DHS) variants (*N*=3,262,787) and 1.1 × 10^−9^ for other non-DHS variants (*N*=16,848,439).

In our data, 37 variants, all located at chr8:129,705,470–129,726,726 (hg38), associated significantly with LDHsurg. We performed conditional analysis for all variants with info>0.9 that are positioned within ±2 Mb of these 37 variants, with rs6651255[C] the variant with the most significant association, as a covariate. No variants in the area had a significant adjusted *P* value (all adjusted *P*>2.3 × 10^−4^).

Logistic regression was performed for LDHsurg case–control status, adjusting for gender and county of origin, with height in terms of SD in cm as explanatory variable (*N*_case_=1,929; *N*_control_=79,883).

To generate height polygenic risk scores, GWAS results for 2,550,858 variants were computed based on GIANT^37^ data after having removed Icelandic samples. Imputed genetic variants were available for 3,085 adult LDHsurg cases and 132,822 controls. Of the GWAS variants which were also available in the imputation set, 1,746,527 were biallelic SNPs and met quality control standards. LDpred^63^ was used to adjust for the correlation among the effects of these variants due to linkage disequilibrium and these adjusted effects were used to produce the adult height polygenic score. Logistic regression was performed for LDHsurg case–control status, adjusting for gender and county of origin, with the polygenic score as explanatory variable. To put the odds ratios for one unit increase in polygenic score in terms of increase in ten cm, we ran a linear regression for adult height in terms of SD in cm with the polygenic score as explanatory variable (*N*=80,546). The regression slope for the polygenic score (*β*=5.5, *P*<1 × 10^−300^) was then used to estimate how many units of the polygenic score would increase height by 10 cm (1.8=10/5.5 units).

### Data availability

Data supporting the findings of this study are available within the article and its Supplementary Information files. Summary level data of markers tested for association is described in Scientific data as of 2015 with the identifier http://dx.doi.org/10.1038/sdata.2015.11 (ref. 64). Whole-genome sequencing summary data are available at the European Variant Archive (EVA, https://www.ebi.ac.uk/eva/) under the accession code PRJEB8636.

GTEx data (http://www.gtexportal.org) accessed 8 August 2016; LD Score Database (ftp://atguftp.mgh.harvard.edu/brendan/1k_eur_r2_hm3snps_se_weights.RDS) accessed 23 June 2015; GWAS catalogue (https://www.ebi.ac.u/gwas/home) accessed 8 September 2016.

## Additional information

**How to cite this article:** Bjornsdottir, G. *et al*. Sequence variant at 8q24.21 associates with sciatica caused by lumbar disc herniation. *Nat. Commun.* **8,** 14265 doi: 10.1038/ncomms14265 (2017).

**Publisher's note:** Springer Nature remains neutral with regard to jurisdictional claims in published maps and institutional affiliations.

## Supplementary Material

Supplementary Information Supplementary Figure, Supplementary Tables, and Supplementary References

## Figures and Tables

**Table 1:** Demographics of cases.

**Phenotype**	***N***	**Age** ***M*** **(s.d.)**	***n*** **with genotypes**	**% with genotypes**
LDHsurg	5,288	44.9 (13.7)	4,748	89.8
Males	3,061	44.3 (13.7)	2,762	90.2
Females	2,227	45.7 (13.7)	1,986	89.2
LDHsurg⩽40 years	2,771	33.1 (6.1)	1,897	68.5

GWAS, genome-wide association studies; LDHsurg, herniated lumbar discs.

Total number of cases undergoing microdiscectomy of an LDHsurg in Iceland 1997–2015 (*N*) and proportion of cases with genotypes included in the GWAS study (*n*).

**Table 2:** GWAS results for variant rs6651255[C] in LDHsurg in Iceland.

**Phenotype**	***N***	**OR**	**95%** **CI**	***P***
LDHsurg	4,748	0.81	(0.77, 0.86)	5.61 × 10^−12^
LDHsurg⩽40yrs*	1,898	0.72	(0.65, 0.79)	3.20 × 10^−11^
LDHsurg>40yrs*	2,850	0.87	(0.81, 0.93)	9.63 × 10^−5^

CI, confidence interval; GWAS, genome-wide association studies; LDHsurg, herniated lumbar discs; OR, odds ratio.

A *χ*^2^-test was used to compute *P* values.

^*^Younger vs older *P*=1.8 × 10^−3^.
