# Supplementary material for: Sequence variant at 8q24.21 associates with sciatica caused by lumbar disc herniation
Source: Nat Commun. 2017 Feb 22;8:14265. doi: 10.1038/ncomms14265 (PMC5322534; doi:10.1038/ncomms14265)
Supplement: Supplementary Information — Supplementary Figure, Supplementary Tables, and Supplementary References [file ncomms14265-s1.pdf]

Sequence variant at 8q24.21 associates with sciatica caused by lumbar disc herniation

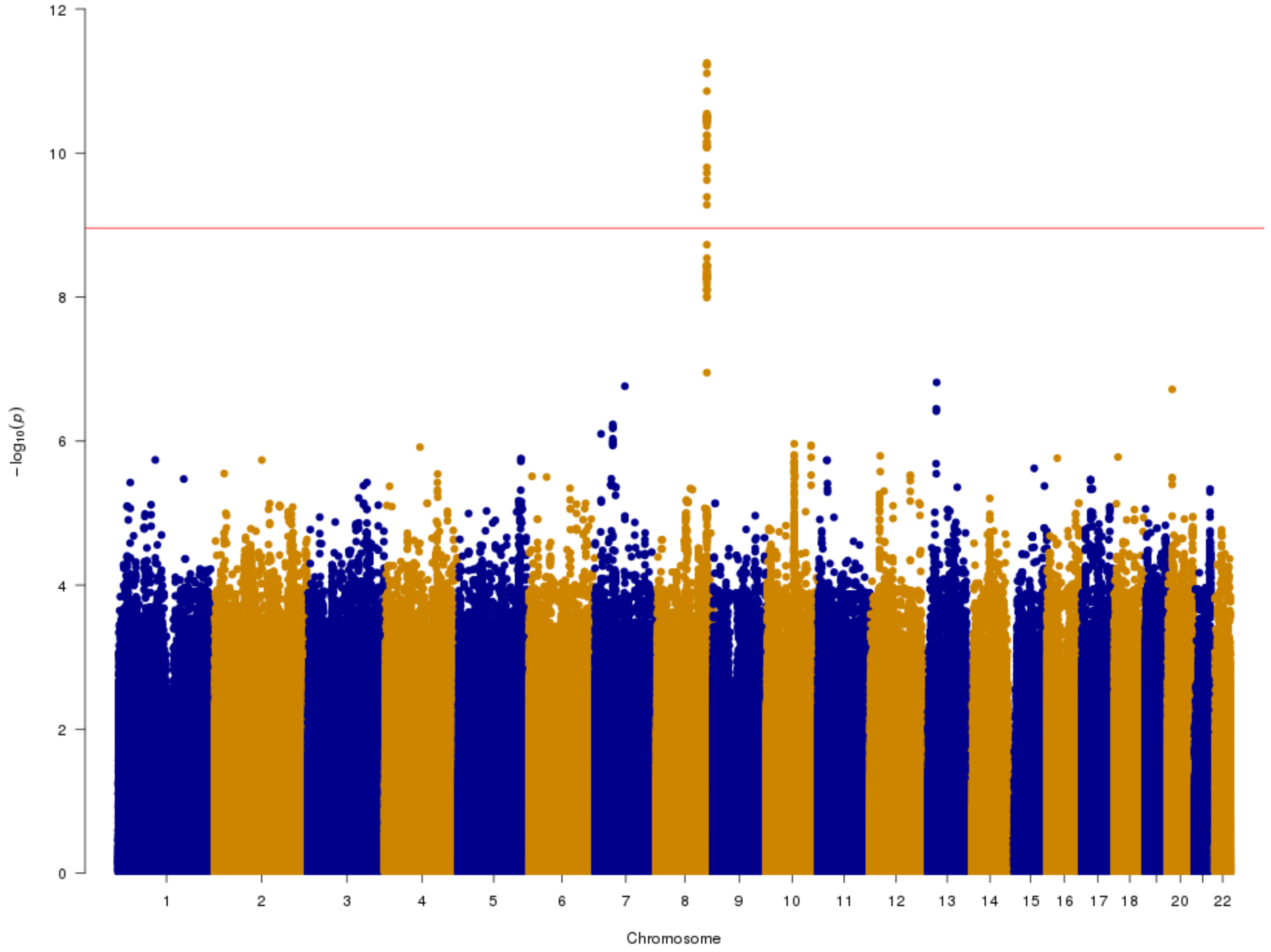

Supplementary Figure 1. Manhattan plot of GWAS findings in the Icelandic discovery sample. A novel locus on 8q24.21 between *GSDMC* and *CCDC26* associated with microdiscectomy for symptomatic herniated lumbar discs (LDHsurg). Additive model. For the plot, the – log10 *P* - values (y axis) of sequence variants are shown according to their chromosomal position (x axis). The red line indicates the weighted threshold[1] for genome-wide statistical significance ($P = 1.1 \times 10^{-9}$). A Chi-squared test was used to calculate *P* - values for all GWAS associations.

marker	Position (hg38)	Effect allele	Effect allele frequency	European allele frequency	Effect (*OR*)	*P*-value
rs6651255	chr8:129711546	C	23.3	21.4	0.813	5.61E-12
rs7833174	chr8:129706526	C	23.3	21.5	0.814	5.97E-12
rs4130415	chr8:129706466	C	23.3	21.5	0.814	5.99E-12
rs7816342	chr8:129707377	A	23.4	21.4	0.815	7.81E-12
rs10956487	chr8:129705470	G	23.2	21.5	0.816	1.38E-11
rs35383270	chr8:129709547	A	21.4	18.6	0.813	2.83E-11
rs6986256	chr8:129716115	A	21.7	6.4	0.815	3.07E-11
rs6470765	chr8:129724451	C	21.7	18.5	0.815	3.09E-11
rs2062078	chr8:129722215	T	21.7	18.5	0.815	3.09E-11
rs4733726	chr8:129719156	T	21.7	18.5	0.815	3.13E-11
rs60228607	chr8:129723214	T	21.7	18.5	0.815	3.13E-11
rs7841981	chr8:129723084	A	21.7	18.7	0.815	3.13E-11
rs3886937	chr8:129725145	C	21.7	18.5	0.815	3.13E-11
rs6985118	chr8:129724683	T	21.7	18.5	0.815	3.14E-11
rs3886938	chr8:129725300	G	21.7	18.5	0.815	3.14E-11
rs373138845, rs66937183	chr8:129721601	G	21.7	18.5	0.815	3.21E-11
rs1074286	chr8:129725355	T	21.7	18.5	0.815	3.23E-11
rs4733724	chr8:129711482	G	21.7	18.7	0.815	3.24E-11
rs7815298	chr8:129720493	C	21.7	13.3	0.815	3.24E-11
rs7826493	chr8:129726726	G	21.7	18.6	0.815	3.48E-11
rs4733732	chr8:129726211	T	21.7	13.8	0.815	3.50E-11
rs10956490	chr8:129718998	C	21.7	18.5	0.815	3.57E-11
rs7815955	chr8:129707321	T	21.7	18.6	0.815	3.64E-11
rs116662391	chr8:129709175	C	21.4	22.56	0.814	3.73E-11
rs6470764	chr8:129713419	T	21.7	18.5	0.816	3.79E-11
rs28743107	chr8:129723416	T	21.7	23.26	0.816	4.21E-11
rs10585686	chr8:129707190	G	21.9	24.35	0.818	5.66E-11
rs79164994	chr8:129707197	G	21.6	24.35	0.817	5.72E-11
rs11984666	chr8:129718034	A	21.6	18.5	0.818	6.95E-11
rs4733725	chr8:129719130	T	21.6	18.5	0.818	7.18E-11
rs10808583	chr8:129705774	A	21.6	18.7	0.818	7.91E-11
rs7814941	chr8:129706613	G	21.6	18.7	0.818	7.95E-11
rs7816131	chr8:129707472	T	21.6	18.6	0.818	8.30E-11
rs35264025,rs59706047	chr8:129709503	T	21.6	18.6	0.818	8.44E-11
rs4368942	chr8:129717272	GA	20.8	21.87	0.818	1.89E-10
rs557156781,rs777595489	chr8:129723419	CT	19.6	0	0.812	2.38E-10
rs6470763	chr8:129708400	C	19.9	18.3	0.819	4.07E-10

Supplementary Table 1. LDHsurg associated markers reaching weighted genome-wide significance threshold[1].

Associations listed in GWAS catalogue with SNPs in *GSDMC* or *CCDC26*											Association of GWAS catalogue SNP with corresponding phenotype in Icelandic data*						Top marker (BestSNP) in region (within +/- 500 Kb of rs6651255) associating with corresponding phenotype in Icelandic data								Association of rs6651255[C] (chr8:129711546) with corresponding phenotype in Icelandic data		
**Phenotype**	**SNP**	**Position (build 38)**	**Close genes**	**Study (PMID), Yr**	**Effect Allele**	**Effect Allele Freq (%)**	***r2* with rs6651255**	**OR**	**Beta**	***P*-value**	***Ncase/ Nctrl*** **[(a)]**	**Effect Allele**	**Effect Allele Freq (%)**	**OR**	**Beta**	***P*-value**	**BestSNP (Pos)**	**Effect Allele**	**Effect Allele Freq (%)**	***r2* with GWAS catalogue SNP**	***r2* of BESTSNP with rs6651255**	**OR**	**Beta**	***P*-value**	**OR**	**Beta**	***P*-value**
**Height**	rs4733724	chr8:129711482	*GSDMC, CCDC26*	Wood AR (25282103), 2014	A	80.2	0.91		0.050	1 E-41	72,613	A	78.3		0.034	1.34E-05	rs7816131 (chr8:129707472)	T	18.6	0.99 (rs4733724), 0.99 (rs6470764)	0.90		-0.036	3.37E-06		-0.032	2.60E-05
	rs6470764	chr8:129713419	*GSDMC, CCDC26*	Lango Allen H (20881960), 2010	T	20	0.91		-0.050	2 E-28	72,613	T	21.7		-0.03	1.35E-05											
**Glioma**	rs4295627	chr8:129673211	*GSDMC, CCDC26*	Sanson M (21531791), 2011	G	NR	0.01	1.40		5 E-21	702/ 358,789	G	17.8	1.10		0.225	rs55862293 [(c)] (chr8:129579134)	G	8.3	0.22 (rs4295627)	0.01	1.71		5.38E-08	1.15		0.069
	rs4295627	chr8:129673211	*GSDMC, CCDC26*	Shete S (19578367), 2009	G	17[(b)]	0.01	1.36		2 E-18																	
**Monocytes**	rs10098310	chr8:129601368	*GSDMC, CCDC26*	Nalls MA (21738480), 2011	A	NR	0.03		0.034	3 E-20	251,559	A	40.4		0.076	8.34E-100	rs10107630 (chr8:129591389)	C	40.3	0.99 (rs10098310)	0.03		0.077	8.22E-101		0.017	4.26E-05
**Orofacial clefts**	rs987525	chr8:128933908	*GSDMC, PVT1*	Birnbaum S (19270707), 2009	A	22.4	< 0.01	2.57		3 E-24	350/ 320,436	A	20.7	1.77		2.32E-06	rs6470759 (chr8:129690034)	G	38.2	< 0.01 (rs987525)	< 0.01	0.68		6.46E-04	0.76		0.038

Supplementary Table 2. Association listed in GWAS catalogue (see URLs) with SNPs in *GSDMC* or *CCDC26*.

NR=Not reported. (a) *N* = cases only for quantitative traits. (b) Effect allele frequency is reported 83% in GWAS catalogue and the original publication states that the ancestral allele frequency is 17%. According to SNP database, G is the ancestral allele, but the original publication seems to assume that the T allele is the ancestral allele. As a result, the GWAS database entry of 83% for the G allele is probably an error, hence we use 17% in the table above.

(c) Comparable results observed for correlated SNP rs55705857 as reported in Jenkins et al., 2012[2].

*Existing in-house data obtained for deCODE studies approved by the Icelandic Ethics Committee and Data Protection Authority.

SNP	AF (%)	*N*	Population	Effect (SD)	*P*	PMID
rs4733724[G]	19.8	252,637	European	- 0.05	1.4×10^{-41}	25282103[3]
rs6470764[T]	19.2	183,727	European	- 0.05	1.7×10^{-28}	20881960[4]
rs2062078[T]	73.0	93,926	East Asian	- 0.04	1.3×10^{-13}	25429064[5]

Supplementary Table 3. Previously reported adult height variants that are highly correlated with rs6651255 [C] ($r^2 > 0.9$) and their association with adult height in large meta-analyses.

Phenotype	Effect (SD)[a]	OR [b]	*P*	N_{case}	$N_{control}$
Osteoarthritis of spine		0.96	0.35	2,009	223,272
Bone mineral density of spine	-0.014		0.31	20,637	
Osteoporosis w/vertebral fractures		1.02	0.64	1,262	178,728

Supplementary Table 4. Effect of rs6651255[C] on selected skeletal phenotypes in Icelandic data[6].

				Williams et al LDD GWAS (*N*=4,600)				Icelandic LDHsurg (N=4,748)				
nr	SNP	Position (build 38)	Gene	Eff all	Beta	SE	*P*-value	Min	Maj	Minor AF (%)	OR	*P*
1	rs2187689	chr6:32884870	-	C	0.23	0.041	2.72E-08	C	T	7.5	1.053	0.27
2	rs7767277	chr6:32885265	-	A	0.23	0.041	2.81E-08	A	C	7.5	1.053	0.27
3	rs926849	chr6:161740587	PARK2	C	-0.13	0.024	3.25E-08	C	T	31.5	1.014	0.59
4	rs7744666	chr6:32891935	-	C	0.2	0.037	5.58E-08	C	T	9.7	1.049	0.25
5	rs11969002	chr6:32891971	-	A	0.2	0.037	5.59E-08	A	G	9.7	1.049	0.25
6	rs6457690	chr6:32887940	-	A	0.19	0.036	9.36E-08	A	G	9.7	1.048	0.25
7	rs1029296	chr6:32888604	-	C	0.19	0.036	9.39E-08	C	T	9.7	1.048	0.25
8	rs6936004	chr6:32889157	-	C	0.19	0.036	1.04E-07	C	T	9.7	1.048	0.26
9	rs3749982	chr6:32894830	-	A	0.19	0.037	1.46E-07	A	T	9.7	1.048	0.26
10	rs9469300	chr6:32892975	-	A	0.19	0.037	1.47E-07	A	G	9.7	1.048	0.25
11	rs10214886	chr6:32889642	-	A	0.19	0.036	2.32E-07	A	T	9.7	1.048	0.25
12	rs10046257	chr6:32886920	-	A	0.19	0.037	3.22E-07	A	G	10.1	1.047	0.26
13	rs3019449	chr6:161744061	PARK2	A	-0.12	0.023	3.68E-07	A	C	31.6	1.011	0.67
14	rs1029295	chr6:32888705	-	C	0.19	0.038	4.58E-07	C	T	9.7	1.048	0.25
15	rs9301951	chr13:94300578	GPC6	C	-0.26	0.054	9.47E-07	C	T	3.8	0.969	0.63
16	rs7896691	chr10:3112981	PFKP	C	0.17	0.035	2.02E-06	C	T	12.0	0.907	0.01
17	rs6602024	chr10:3113045	PFKP	A	0.17	0.035	2.20E-06	A	G	12.0	0.907	0.01
18	rs17132175	chr10:3108622	PFKP	C	0.16	0.036	3.73E-06	C	G	12.0	0.907	0.01
19	rs737631	chr6:161741401	PARK2	A	-0.11	0.025	4.32E-06	A	G	31.4	1.01	0.70
20	rs4802666	chr19:50217817	MYH14	A	-0.13	0.029	4.55E-06	A	G	31.4	0.997	0.91
21	rs1205863	chr6:11943293	-	G	0.21	0.047	5.64E-06	G	T	11.7	1.036	0.35
22	rs11918654	chr3:5146561	ARL8B	C	-0.11	0.024	7.10E-06	C	T	28.8	0.942	0.03
23	rs4881085	chr10:3113348	PFKP	A	0.16	0.036	7.44E-06	A	G	12.0	0.906	0.01
24	rs12805875	chr11:103658904	-	A	0.09	0.021	8.51E-06	A	G	40.2	1.023	0.37
25	rs7103004	chr11:103655296	-	C	0.09	0.021	9.20E-06	C	T	40.2	1.023	0.37
26	rs4554859	chr11:103659638	-	G	0.09	0.021	9.24E-06	G	T	40.2	1.023	0.37
27	rs7118412	chr11:103655026	-	A	0.09	0.021	9.37E-06	A	G	40.2	1.022	0.38
28	rs17034687	chr3:3638168	-	G	-0.23	0.038	1.82E-09	G	C	9.2	1.016	0.71
29	rs4875102	chr8:4427170	-	G	0.12	0.024	3.61E-07	G	A	28.4	0.989	0.69
30	rs1884158	chr6:161740634	PARK2	T	0.11	0.023	3.56E-06	T	C	31.0	1.014	0.59
31	rs10998466	chr10:68866673	-	G	0.53	0.114	3.59E-06	G	A	0.8	0.896	0.44
32	rs1981483	chr16:580665	PIGQ	G	-0.11	0.023	3.75E-06	G	A	46.6	0.98	0.43
33	rs1154053	chr8:4427868	-	G	0.13	0.028	3.99E-06	G	C	20.1	1.005	0.86
34	rs2484990	chr10:31226203	-	T	-0.68	0.148	4.39E-06	T	C	0.1	1.507	0.29
35	rs1250307	chr10:31207045	-	G	-0.68	0.148	4.39E-06	G	A	0.1	1.505	0.29
36	rs7204439	chr16:611335	RAB40C	T	-0.11	0.023	4.43E-06	T	C	45.9	0.985	0.55
37	rs2484992	chr10:31223169	-	T	-0.68	0.149	4.81E-06	T	C	0.1	1.507	0.29
38	rs9488238	chr6:113695931	-	G	0.28	0.061	5.17E-06	G	A	3.5	0.959	0.54
39	rs763014	chr16:625680	RAB40C	T	-0.1	0.023	5.43E-06	T	C	44.2	0.977	0.35
40	rs2657195	chr8:91547687	-	G	0.13	0.028	7.54E-06	G	A	18.0	0.99	0.74
41	rs11754641	chr6:64926030	-	G	-0.29	0.064	7.84E-06	G	C	4.9	1.001	0.99

				Williams et al LDD GWAS (*N*=4,600)				Icelandic LDHsurg (N=4,748)				
nr	SNP	Position (build 38)	Gene	Eff all	Beta	SE	*P*-value	Min	Maj	Minor AF (%)	OR	*P*
42	rs980238	chr8:4425096	-	C	0.1	0.023	9.11E-06	C	A	30.7	0.981	0.47
43	rs2017567	chr16:587212	-	T	-0.1	0.021	9.38E-06	T	C	46.6	0.98	0.42
44	rs710924	chr16:583353	PIGQ	T	-0.09	0.021	9.93E-06	T	C	46.6	0.981	0.45

Supplementary Table 5. Association of markers from Williams *et al.* (2013)[7] with the morphologically defined LDD phenotype, and with the symptomatically determined LDHsurg phenotype in Icelandic data.

Phenotype	OR	P	N_{case}	$N_{control}$
Chronic pain conditions				
Temporomandibular joint disorder	0.99	0.87	1,455	307,435
Fibromyalgia	0.94	0.20	2,142	283,446
Migraine	0.98	0.62	3,816	289,201
Neuropathic pain				
Neuropathic pain (DN4)	0.83	0.04	394	220,531

Supplementary Table 6. Effect of rs6651255[C] on chronic and neuropathic pain phenotypes in Icelandic data.

		LDHSurg		*GSDMC*				*RP11-274M4.1*	
		Association results		*Esophagus-Mucosa*		*Skin-Sun exp. (lower leg)*		*Esophagus-Mucosa*	
SNP ID	***r^2 with rs6651255***	***P***	**OR**	***P***	**Effect**	***P***	**Effect**	***P***	**Effect**
rs6651255	-	$5.6×10^{-12}$	0.81	$9.2×10^{-14}$	0.60	$4.5×10^{-10}$	0.31	$3.7×10^{-6}$	-0.39
rs4345520	0.29	0.06	1.05	$3.0×10^{-42}$	0.89	$3.3×10^{-28}$	0.45	$2.7×10^{-15}$	-0.59
rs10092783	0.24	0.11	1.04	$7.9×10^{-18}$	0.66	$3.6×10^{-18}$	0.36	$6.7×10^{-16}$	-0.63

Supplementary Table 7. Significant eQTLs of rs6651255 and the variants that have the most significant eQTLs in the same tissues: rs4345520 and rs10092783 (GTEx, anlysis release V6, see URLs).

Phenotype	*N*	Age *M* (*SD*)
M51.1 w/o LDHsur	930	47.2 (14.3)
Males	512	47.1 (15.2)
Females	418	47.2 (13.2)

Supplementary Table 8. Demographics of cases with a diagnosed herniated lumbar disc (ICD-10 code M51.1) in Iceland 1997-2015 (N=930), who did not undergo LDHsurg.

Supplementary References

1. Sveinbjornsson, G. *et al.* Weighting sequence variants based on their annotation increases power of whole-genome association studies. *Nat Genet* **48**, 314-7 (2016).
2. Jenkins, R.B. et al. A low-frequency variant at 8q24.21 is strongly associated with risk of oligodendroglial tumors and astrocytomas with IDH1 or IDH2 mutation. Nat Genet 44, 1122-5 (2012).
3. Wood, A.R. *et al.* Defining the role of common variation in the genomic and biological architecture of adult human height. *Nat Genet* **46**, 1173-86 (2014).
4. Lango Allen, H. *et al.* Hundreds of variants clustered in genomic loci and biological pathways affect human height. *Nature* **467**, 832-8 (2010).
5. He, M. *et al.* Meta-analysis of genome-wide association studies of adult height in East Asians identifies 17 novel loci. *Hum Mol Genet* **24**, 1791-800 (2015).
6. Styrkarsdottir, U. *et al.* Sequence variants in the PTCH1 gene associate with spine bone mineral density and osteoporotic fractures. *Nat Comm* **7**, 10129 (2016).
7. Williams, F.M. *et al.* Novel genetic variants associated with lumbar disc degeneration in northern Europeans: a meta-analysis of 4600 subjects. *Ann Rheum Dis* **72**, 1141-8 (2013).
